# Exercise Similarly Facilitates Men and Women’s Selective Attention Task Response Times but Differentially Affects Memory Task Performance

**DOI:** 10.3389/fpsyg.2018.01405

**Published:** 2018-08-13

**Authors:** Matt Coleman, Kelsey Offen, Julie Markant

**Affiliations:** Department of Psychology, Tulane University, New Orleans, LA, United States

**Keywords:** acute exercise, sex differences, selective attention, episodic memory, recognition memory

## Abstract

Previous research has found that acute, moderate-intensity physical exercise enhances selective attention and memory and that men and women show differential performance on tasks measuring these skills. Although exercise and participant sex have been examined separately, it remains unknown whether acute, moderate-intensity exercise differentially affects men and women’s selective attention and memory encoding and retrieval. Participants in the present study completed two 10-min sessions of either moderate-intensity exercise comprised of jumping rope alternating with walking in place or an active control protocol comprised of watching wellness videos alternating with walking in place. Each participant completed a selective attention task and a task assessing recognition and object location memory immediately after exercising. Exercise was related to overall faster performance during the selective attention task, with no differences in men and women’s performance. Women showed better recognition memory compared to men. Exercise specifically improved object location memory among men, but only among participants who completed the memory task second. These findings suggest that acute, moderate-intensity exercise differentially affects men and women’s memory, which may be related to complex interactions between exercise, sex hormones, and the neurotrophin BDNF.

## Introduction

Researchers are increasingly recognizing that exercise has important benefits for both physical fitness and cognitive performance during adulthood (Prakash et al., 2015). In particular, this work has shown that even short bouts of moderate-intensity exercise results in improved performance on measures of executive functions, attention, and memory (Lambourne and Tomporowski, 2010). However, although prior research has found sex differences in both selective attention (Van der Elst et al., 2006; Stoet, 2010; Evans and Hampson, 2015) and memory performance (Herlitz and Rehnman, 2008), little research has examined whether moderate-intensity acute exercise differentially affects cognitive performance among men and women. The present study addressed this question by assessing male and female participants’ selective attention, recognition memory, and object location memory performance immediately following two 10-min bouts of moderate-intensity exercise.

Previous research has shown that habitual exercise can protect against cognitive decline among older adults (Prakash et al., 2015) and supports improved executive function and memory skills among young adults (Gaertner et al., 2017). Individuals who demonstrate higher levels of aerobic fitness similarly perform better on measures of memory, attention, and inhibitory control (Åberg et al., 2009; Scott et al., 2016; Blumenthal et al., 2017; Ciria et al., 2017; Schwarb et al., 2017). Randomized controlled trials have further shown that long-term exercise training programs enhance cognitive function among healthy adults and mitigate cognitive decline among older adults, including those with and without cognitive impairment (Colcombe and Kramer, 2003; Etnier et al., 2006; Guiney and Machado, 2013). Furthermore, studies with older adults have shown that exercise training was associated with increased volume of brain regions that support selective attention and memory, including the hippocampus and anterior cingulate cortex (Colcombe et al., 2006; Erickson et al., 2011). Far fewer studies have examined long-term aerobic exercise interventions among healthy young adults, with mixed results indicating improvements in only some aspects of memory and executive functions (Stroth et al., 2009, 2010; Gomes-Osman et al., 2017).

More recent work has examined whether shorter bouts of exercise can similarly improve cognitive skills among healthy young adults. This research has shown that acute, moderate-intensity aerobic exercise interventions promote executive function, attention, and memory skills (Joyce et al., 2009; Pontifex et al., 2009; McMorris and Hale, 2012; Nanda et al., 2013; Alves et al., 2014; Akatsuka et al., 2015; Basso et al., 2015; Perini et al., 2016; Samani and Heath, 2018). Multiple studies have used the Eriksen Flanker task (Eriksen and Eriksen, 1974) to examine the effects of acute exercise on selective attention. In this task, individuals are asked to indicate the direction of a central target arrow that is surrounded by multiple distractors that point in either the same direction (*congruent* trials) or the opposite direction (*incongruent* trials). Incongruent trials require increased selective attention to suppress the conflicting distractor information and accurately indicate the direction of the central target arrow. Previous work has shown that 20–60 min of moderate- or high-intensity exercise either during or immediately prior to completing this task resulted in overall faster response times (Kamijo et al., 2007; Davranche et al., 2009; Kao et al., 2017; Tempest et al., 2017). However, this response facilitation did not vary across congruent and incongruent trial types (Kamijo et al., 2007; Davranche et al., 2009), suggesting that the exercise had a more general effect on participants’ processing speed/motor responses.

Acute, moderate-intensity exercise has also shown benefits for young adults’ long-term memory encoding and retrieval. Several studies have shown that 10–40 min of moderate-intensity aerobic exercise supported improved memory recall (Coles and Tomporowski, 2008; Labban and Etnier, 2011; Sng et al., 2017), procedural memory and recognition memory (McNerney and Radvansky, 2015), and memory discrimination (Suwabe et al., 2017). Suwabe et al. (2017) found that 10 min of moderate exercise (i.e., 50% peak VO_2_) prior to encoding novel images specifically improved subsequent discrimination of old vs. new images in the hardest condition, when images were most similar to each other. Finally, a recent meta-analysis indicated that acute exercise interventions had an overall moderate to large effect on long-term memory processes and that encoding immediately after the exercise intervention conferred the greatest benefits for subsequent memory (Roig et al., 2013).

Researchers have identified a number of possible mechanisms underlying these improvements in memory following exercise, including neurovascular changes (Endres et al., 2003; Pereira et al., 2007), increased release of neurotrophins (Neeper et al., 1995), or increased neurogenesis in regions of the hippocampus (van Praag et al., 2002). Extensive research with animal models has shown that hippocampal neurogenesis can be regulated by a number of extrinsic factors, including cardiovascular exercise (Farioli-Vecchioli and Tirone, 2015; Saraulli et al., 2017). Conversely, restricting exercise through hindlimb suspension resulted in reduced hippocampal neurogenesis, even after the restraint was removed (Yasuhara et al., 2007). Animal models have also shown that the enhancement of hippocampal neurogenesis via exercise in turn promotes enhanced performance on hippocampal-dependent learning and memory tasks (Creer et al., 2010; Kohman et al., 2012).

Researchers are also increasingly recognizing that participants’ sex may have a moderating influence on the relationship between exercise interventions and changes in cognitive function. Emerging research has demonstrated differential performance for men and women during both selective attention and memory tasks (Van der Elst et al., 2006; Herlitz and Rehnman, 2008; Stoet, 2010; Heisz et al., 2013; Evans and Hampson, 2015; Barha et al., 2017; Sattari et al., 2017). Multiple studies have found that women performed worse on the Flanker selective attention task relative to men, showing increased slowing of response times and/or reduced accuracy on trials with high conflict (i.e., incongruent trials) (Stoet, 2010; Judge et al., 2012; Evans and Hampson, 2015). Clayson et al. (2011) further demonstrated differential neural processing during the Flanker task, as men showed a slower latency and larger amplitude event-related-potential (i.e., ERP) that is typically associated with resolving conflict (e.g., N2). Although this research suggests a consistent performance advantage for men during the Flanker task, research using the Stroop task, which similarly requires selective attention to resolve interference among competing stimuli, has shown more mixed results. Specifically, some researchers have observed no performance differences across men and women (Daniel et al., 2000) while other studies have found a performance advantage for women over men (Mekarski et al., 1996; Van der Elst et al., 2006).

Unlike these mixed results for selective attention, previous research has found that women reliably perform better than men on measures of recognition memory, including those assessing memory for faces (Heisz et al., 2013; Sattari et al., 2017), objects (McGivern et al., 1997, 1998), and verbal information (Herlitz et al., 1997; Lewin et al., 2001). Research has also shown that women outperform men on spatial memory tasks that require remembering the location of previously encoded objects (Silverman and Eals, 1992; Eals and Silverman, 1994). In a meta-analysis of thirty-six studies, Voyer et al. (2007) found that women performed significantly better than men in the majority of these object location memory tasks. This advantage in memory performance among women may be mediated by sex hormones. Research with animal models has shown that direct administration of estradiol supports improvements in both spatial and object recognition (see Choleris et al., 2018 for review) and performance on attention tasks varies based on hormonal changes associated with the menstrual cycle (Pletzer et al., 2014). However, the observed differences in memory performance among men and women may also be related to language processing skills. Consistent with this, women showed better recognition memory on a series of verbal memory tasks, whereas men showed better memory for visuospatial information that could not be easily labeled (Lewin et al., 2001). Women also perform better than men on object location memory tasks when the target objects can be easily identified with a verbal label (Voyer et al., 2007) but this effect was attenuated when the targets were abstract objects that could not be easily labeled (Rahman et al., 2011).

Few studies have examined whether exercise differentially affects cognitive function among men and women, despite this emerging evidence for baseline differences in selective attention and memory performance. The majority of studies that have addressed this question have focused on long-term exercise interventions with older adults and/or patient populations. An early meta-analysis of randomized exercise interventions on older adults’ cognitive skills, including cognitive control, executive functions, and processing speed, found that studies with a higher proportion of women showed significantly larger effects (Colcombe and Kramer, 2003). A more recent meta-analysis of long-term aerobic exercise interventions with middle-aged and older adults similarly found that studies with a higher proportion of women showed stronger effects on executive functions but no difference in effect sizes for memory (Barha et al., 2017). Two studies have directly examined differential effects of exercise interventions on cognitive functions among men and women. Baker et al. (2010) assessed selective attention and memory performance among adults with mild cognitive impairment before and after a 6-month high-intensity aerobic exercise intervention. The exercise intervention specifically improved selective attention performance among women and had no effect on memory performance for either men or women (Baker et al., 2010). Finally, Most et al. (2017) found that 5 min of acute exercise following memory encoding improved healthy young women’s paired associate memory but had no effect on men’s memory performance.

This research suggests that acute exercise benefits cognitive functioning and that exercise may differentially affect selective attention and memory among men and women. However, these findings remain mixed due to variability in the timing, nature, and intensity of the exercise protocols used across studies. To our knowledge only one study (Most et al., 2017) has specifically examined whether acute exercise differentially influenced memory among men and women, despite growing evidence for sex differences in both selective attention and memory performance. The present study thus examined whether acute, moderate-intensity aerobic exercise differentially affected selective attention and/or memory among healthy, young adult men and women. The exercise protocol consisted of five 1-min bouts of jumping rope at a moderate intensity alternating with five 1-min bouts of low-intensity walking in place. We used an active control group to correct for potential expectation-driven placebo effects on cognitive outcomes within the exercise group (Alves et al., 2014; Weng et al., 2015). This active control protocol consisted of five 1-min bouts of watching a video alternating with five 1-min bouts of low-intensity walking in place (c.f., Alves et al., 2014). Thus participants in both the exercise and control groups condition engaged in some activity and were unaware of their group placement. All participants completed the exercise/control protocol twice, once before completing a selective attention task and again before completing a memory task. We used the Flanker task described above to assess selective attention. To assess memory we utilized an episodic memory task modeled after DeMaster et al. (2013), which found increased hippocampal activity associated with accurate object location memory among adults. Use of this task allowed us to examine recognition memory for specific target objects as well as spatial memory for the location of the target objects seen during encoding.

We predicted that acute exercise would facilitate performance on both the selective attention and memory tasks. We expected that participants in the exercise group would demonstrate overall faster response times during the selective attention task, regardless of distractor congruency, and better recognition and object location memory. We also expected that women would show poorer selective attention, indicated by increased slowing of response time during incongruent trials, but better recognition and object location memory performance. Finally, we expected that exercise may differentially influence men and women’s selective attention and memory performance, with exercise offering more benefit for the sex that typically shows a performance disadvantage relative to the other sex. Thus, we expected that women’s selective attention performance and men’s memory performance may show the greatest benefit from the exercise intervention.

## Materials and Methods

### Participants

The final sample included 52 adults (25 Male; *M*_age_ = 19.7 years, *SD* = 1.2 years). This sample size is consistent with previous studies examining the effects of exercise on cognition (see Roig et al., 2013, for review). According to self-report, 82.7% of participants were White, 7.7% were Hispanic, 5.8% were Black, and 3.8% were multiracial. Participants were recruited from the community through advertisements or through the university’s online recruitment system for course credit. Individuals were excluded from participating in the study if they were over 35 years of age, had a history of neurological or developmental disorders (e.g., Down’s Syndrome, Fetal Alcohol Syndrome, Autism Spectrum Disorders, Attention Deficit Hyperactivity Disorder), uncorrected visual impairments, or a Body Mass Index of 40 or above. All participants received either payment or course credit for completing the study. Eight additional participants were tested but excluded due to technical problems with the stimulus presentation software (5), failure to complete the full experimental session (1), a diagnosis of Attention Deficit/Hyperactivity Disorder (1), or an IQ score that was >3 *SD* below the group mean (1, see section “IQ Assessment” for details). This low IQ score may reflect altered global cognitive functioning, difficulty following the researcher’s instructions, or general disengagement from the experimental session. All study procedures were approved by the local Institutional Review Board and participants provided informed consent prior to completing the study.

### IQ Assessment

Participants completed the Vocabulary and Matrix Reasoning subtests of the Wechsler Abbreviated Scale of Intelligence (WASI) (Wechsler, 2011). The two subtest version provides a full-scale IQ score that summarizes the individual’s global cognitive functioning.

### Apparatus

#### Heart Rate Monitor

We used a FitBit Charge HR Wireless Activity Wristband (Model: FB405BKL) to monitor participants’ heart rates during exercise. The FitBit was Bluetooth-enabled allowing the researcher to view heart rates in real time on a remote screen. The FitBit monitored participants’ heart rate continuously throughout each of the 10-min activity protocols (see “Exercise Protocol”). A researcher used a stopwatch to monitor timing throughout the activity protocols.

#### Stimulus Presentation

Participants sat approximately 60 cm from a 24″ LED monitor (Dell U2415) with 1920 × 1080 resolution. All stimuli were presented using E-Prime 2.0 (Psychology Software Tools, Pittsburgh, PA, United States).

### Stimuli

#### Selective Attention Task

**FIGURE 1:**
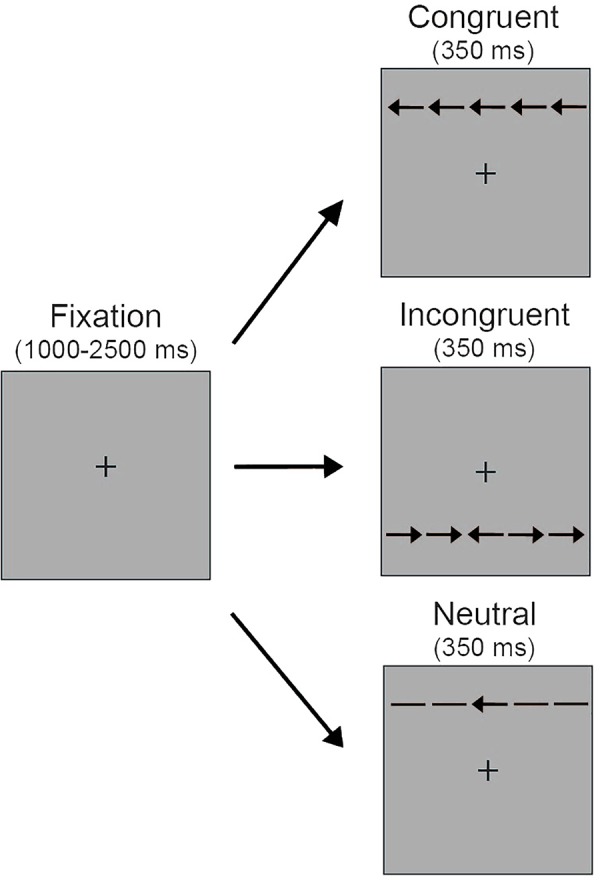
Schematic depiction of the selective attention task. Five arrows appeared either above or below the central fixation on a gray background. Participants were instructed to look at the central fixation and press a keyboard button corresponding to the orientation of the center arrow. During neutral trials, the distractors surrounding the center target arrow were non-directional horizontal lines. During congruent trials the surrounding distractors were in the same direction as the center target arrow (i.e., left target arrow, left distractor arrows; right target arrow, right distractor arrows). During incongruent trials the surrounding distractors were in the opposite direction of the center target arrow (i.e., left target arrow, right distractor arrows; right target arrow, left distractor arrows).

Stimuli for the selective attention task comprised a central target arrow (0.55° visual angle) surrounded by four identical distractors of equivalent size, with two distractors appearing to the left of the target and two distractors appearing to the right of the target (**Figure 1**). Distractors were spaced 0.06° apart. Distractors were arrows pointing in the same direction as the target arrow (congruent trials), arrows pointing in the opposite direction (incongruent trials) or horizontal lines (neutral trials). Thus the full stimulus set was a single row of five black arrows (3.08°). The arrows were presented 1.0° above or below a central fixation cross (0.3°). Stimuli were presented on a gray background.

#### Memory Task

**FIGURE 2:**
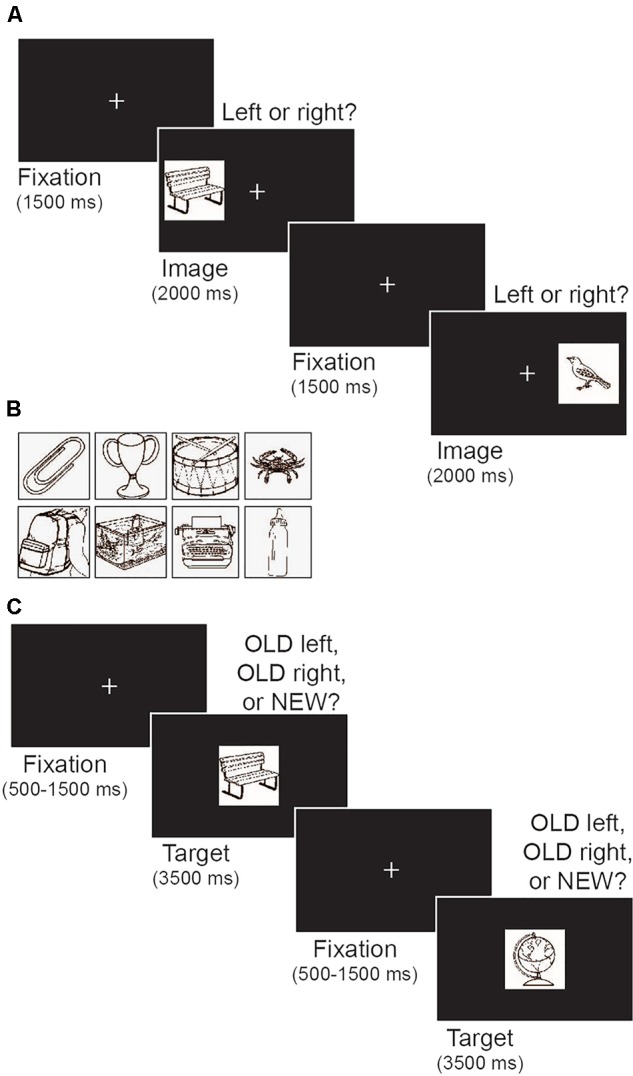
Illustration of the memory task. **(A)** Schematic depiction of the encoding phase. Participants were told to memorize the target image and its left/right location. **(B)** Examples of images of everyday objects that were used as targets. **(C)** Schematic depiction of the test phase. Participants indicated whether each target image was old or new, and if old, whether the image appeared on the left or right during encoding.

Stimuli were presented on a black background and included a white fixation cross (0.9° × 1.0°) and a set of target objects (4.8° × 5.3°; **Figure 2A**). Targets were black and white images of everyday objects selected from the International Picture Naming Project database (Szekely et al., 2004; **Figure 2B**). Two sets of 100 objects were used. The first set was used as target objects during the encoding phase of the memory task and reappeared as old items during the subsequent test phase. The second set provided novel objects during the memory test phase.

### Procedure

#### Exercise Protocol

**FIGURE 3:**
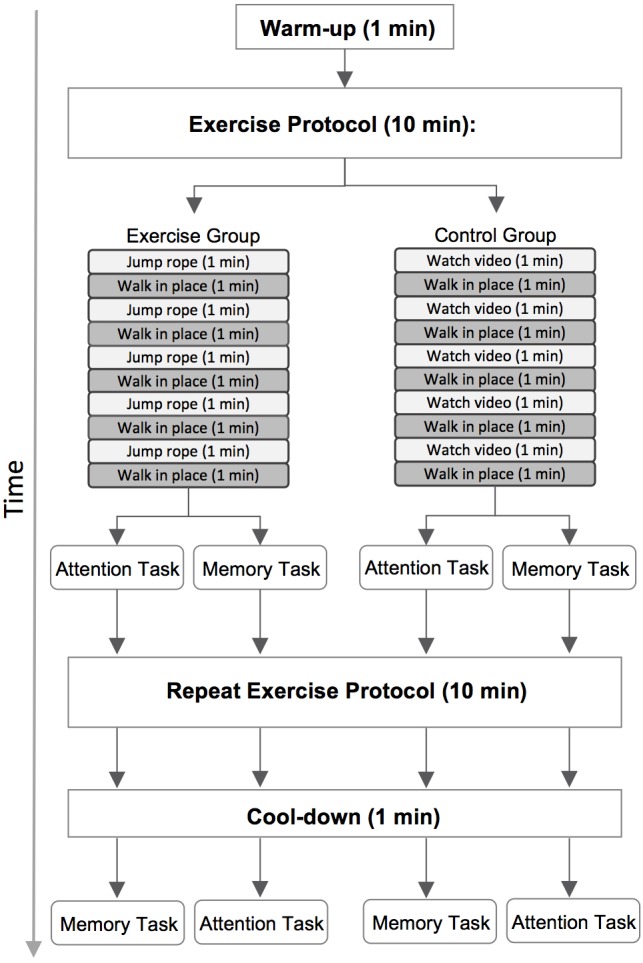
A diagram of the full test session. Participants performed a 10-min exercise or control activity, depending on group placement, before each of the two computer tasks. Half of the participants completed the attention task first and the memory task second, and the remaining participants completed the tasks in the reverse order.

We placed the FitBit on participants wrists at the beginning of the test session and measured resting heart rate while they completed the TestWell Holistic Lifestyle Questionnaire (National Wellness Institute, 1992), an online questionnaire designed to measure different categories of wellness. Data from this questionnaire were not analyzed for the present study. All participants completed two 10-min activities prior to each of the two computer tasks (**Figure 3**). Participants were randomly assigned to either the exercise or the control group. Participants were shown a visual relative perceived exertion (RPE) scale depicting varying degrees of physical exertion to give them a frame of reference for low-intensity exertion (e.g., 2 out of 10) versus moderate-intensity (e.g., 6 out of 10).

Participants in both groups began by walking in place for 1 min to warm up. Participants in the exercise group were then instructed to jump rope at a moderate-intensity pace for 1 min, followed by 1 min of walking in place at a low-intensity pace. This sequence was repeated four more times for a total of 10 min of activity. Participants began the first computer task immediately after completing the first 10-min exercise protocol. They then completed a second identical exercise protocol. After completing this second round of exercise participants walked in place for one additional minute to cool-down. This cool-down period ensured that the total duration of activity was equated across the first and second exercise protocols (11 min total). Participants immediately began the second computer task after completing the cool-down.

We used an active control design to ensure that participants would be unaware whether they had been randomized into the exercise or control group. Participants in the control group thus completed a very similar protocol. Participants again began with a 1 min warm up period and then first viewed a brief video describing healthy lifestyle habits for 1 min, followed by 1 min of walking in place at a low-intensity pace. This sequence was repeated four times, for a total of 10 min of activity. As in the exercise group, participants in the control group began the first computer task immediately after completing the first activity protocol. They then completed a second identical 10-min activity protocol followed by an 1-min cool down period of walking in place. Participants began the second computer task immediately after finishing the cool down.

A researcher monitored the timing of the activity protocols and indicated when participants should begin jumping rope or walking in place. The researcher recorded participants’ heart rate with 5 s left of each of the ten 1-min bouts in order to avoid inaccurate measurements that may occur when participants transitioned between activities.

#### Cognitive Tasks

All participants completed both the selective attention and memory tasks. Task order was counterbalanced across participants so that half of the participants completed the selective attention task after the first activity protocol and completed the memory task after the second activity protocol. The remaining participants completed the tasks in the reverse order (memory task first, selective attention task second).

##### Selective attention task

**Figure 1** illustrates the selective attention task. At the beginning of each trial a central fixation cross appeared for a variable duration (1000–2500 ms), which was intended to reduce temporal expectations and increase engagement in the task. Next, the arrow stimuli appeared either above or below the central fixation for 350 ms. Participants were instructed to maintain fixation at the central location while using key presses to indicate if the target arrow pointed to the left or right. Participants had up to 1700 ms to respond. The arrow stimuli remained on the screen for the 350 ms trial duration regardless of response time. There were three possible trial types. Participants completed a total of 180 trials, with 60 neutral, 60 congruent, and 60 incongruent trials presented in random order. The number of trials with arrows presented above and below the central fixation and with the central target pointing to the left and right was equally distributed within each of these trial types.

##### Memory task

The memory task was modeled after DeMaster et al. (2013). The task comprised an initial encoding phase followed by a test phase that assessed both recognition memory and object location memory. **Figure 2A** illustrates the encoding phase of the task. Each trial began with a central fixation that remained on screen for 1500 ms. Next, a target object appeared on the left or right side of the screen (17.3° from center) for 2 s, followed by a variable inter-trial interval (500, 1000, 1500, or 2000 ms). The variable inter-trial interval was a within-subject manipulation intended to prevent participants from making anticipatory responses based on predictable timing between trials. All participants had the same distribution of possible inter-trial interval durations. Participants were instructed to memorize both the target object and its location while using keyboard presses to indicate whether the target appeared on the left or right side. The encoding phase included 100 trials, with an equal number of targets appearing on the left and right (50 each) in random order.

**Figure 2C** illustrates the test phase of the memory task. During each trial a single object image was presented in the center of screen for 3500 ms. A central fixation appeared between each image for a variable duration (500, 1000, or 1500 ms). Participants were instructed to press 1 if the image was old and appeared on the left side of the screen during encoding, press 2 if it was old and appeared on the right side during encoding, or press 3 if it was new. Participants were told to make a response within the 3500 ms in which the image was visible on the screen. As in DeMaster et al. (2013) the test phase comprised 175 trials, including all 100 object images presented during the encoding phase and 75 novel images. Participants were unaware of the proportion of old and new images. Old and new images were presented in random order. Two sets of 100 images were each used for either the encoding phase or as novel images in the test phase. Set order was counterbalanced across participants.

### Data Processing

#### Heart Rate

We computed Heart Rate Reserve percentage (HRR%) as a measure of participants’ exertion based on the formula HRR% = [Heart rate - Rest HR]/[Max HR - Rest HR] (Karvonen and Vuorimaa, 1988). This measure was used instead of raw heart rate to account for individual differences in resting heart rate and maximum heart rate.

#### Selective Attention

For the selective attention task we focused on accuracy and response time to indicate the direction of the central target arrow. We excluded individual trials if the participant failed to make a response (*M* = 2.4% trials, *SD* = 6.1%). When calculating participants’ average response times we only included trials in which they made a correct response.

#### Memory

##### Encoding phase

For the encoding phase of the memory task we focused on accuracy and response time to indicate whether the target object was on the left or right side of the screen. Individual trials were excluded if the participant failed to make a response (*M* = 1.1% trials, *SD* = 1.6%). We again calculated participants’ average response times based only on trials in which the participant made a correct response.

##### Test phase

We computed recognition memory sensitivity scores (*d*′) for each participant based on his/her accuracy in discriminating the old versus new images during the test phase. *D*′ is a common measure of recognition memory sensitivity that is based on signal detection theory (Yonelinas et al., 1996). We computed *d*′ scores by subtracting the z-transformed proportion of false alarms (i.e., incorrectly identifying a new image as old) from the z-transformed proportion of hits (i.e., correctly identifying an old image). Individual trials were excluded if participants did not make a response within the 3500 ms time limit (*M* = 1.2% trials, *SD* = 1.5%).

We also examined participants’ accuracy in identifying the location (i.e., left versus right) in which the old images appeared during the encoding phase of the task. This object location memory analysis was conducted only for images that the participant correctly identified as old. Object location memory accuracy was thus computed as the number of old images for which the correct encoding location was identified divided by the total number of images that were correctly identified as old. Object location memory accuracy was computed separately for old images that appeared in the left and right locations during encoding. Based on these accuracy scores we then computed object location memory sensitivity (object location *d*′). Object location *d*′ scores were computed by subtracting the z-transformed proportion of left false alarms (i.e., incorrectly identifying an image that appeared on the right as having appeared on the left) from the z-transformed proportion of left hits (i.e., correctly identifying images that appeared on the left as having appeared on the left).

## Results

### Preliminary Analyses

We first used a 2 (group) × 2 (sex) ANOVA to examine potential differences in age and IQ across groups. Mean values are presented in **Table 1**. One participant did not complete the WASI, resulting in a sample of *N* = 51 for IQ analyses. Results confirmed that there was no main effect of exercise group on either age, *F*(1,48) = 0.32, *p* = 0.575, or IQ, *F*(1,47) = 0.01, *p* = 0.918. There was also no main effect of sex on age, *F*(1,48) = 0.003, *p* = 0.959, or IQ, *F*(1,47) = 0.57, *p* = 0.455. Finally, the group × sex interaction was not significant for either age, *F*(1,48) = 0.94, *p* = 0.337, or IQ, *F*(1,47) = 0.38, *p* = 0.539. Thus male and female participants in the control and exercise groups were similar with respect to both age and IQ.

**Table 1:** Mean age and IQ scores by exercise group and participant sex.

		Age	IQ
		Mean (*SD*)	
Control	Male	19.49 (*0.79*)	104.58 *(16.87*)
	Female	19.80 *(1.65*)	104.15 (*11.94*)
Exercise	Male	20.01 (*0.99*)	106.23 (*5.12*)
	Female	19.66 (*1.21*)	101.85 (*8.92*)

We also confirmed the efficacy of the exercise protocol. We examined participants’ HRR% scores using an ANOVA with activity protocol (block 1, block 2) as a within subjects factor and group (control, exercise) and participant sex (male, female) as between subjects factors. Results indicated a significant main effect of group, *F*(1,47) = 331.76, *p* < 0.001, η^2^ = 0.88, with higher HRR% in the exercise group (*M* = 39.6%, *SD* = 7.15%) compared to the control group (*M* = 3.0%, *SD* = 7.21%). There was also a significant main effect of participant sex, *F*(1,47) = 4.60, *p* = 0.037, η^2^ = 0.09, with females showing a higher HRR% compared to males (*M*_M_ = 19.2%, *SD* = 7.18%; *M*_F_ = 23.5%, *SD* = 7.16%). This main effect of sex was no longer significant (*p* = 0.06) when we excluded a female participant from the exercise group whose HRR% was >2 *SD* above the group mean; however, the main effect of group remained significant (*p* < 0.001). There was no main effect of block or group x sex interaction [block: *F*(1,47) = 0.82, *p* = 0.369; group × sex: *F*(1,47) = 2.88, *p* = 0.096]. These results confirm that exercise effectively increased participants’ HRR%, relative to the control group, to a similar extent during each of the two exercise protocols.

### Selective Attention Task

We examined accuracy and response times using a mixed ANOVA with trial type (neutral, congruent, and incongruent) as the within-subjects factor and group (exercise, control), sex (male, female), and task order (attention-memory, memory-attention) as between-subjects factors. Greenhouse-Geisser corrections were used as necessary to correct for violations of sphericity. Three participants (1 control male, 2 exercise male) were excluded from all analyses due to a high rate of failing to respond (>3 *SD* above the group mean). Two additional participants (1 male control, 1 female control) were excluded from all analyses due to poor overall accuracy (<3 *SD* below the group mean). These high rates of failing to respond and committing errors suggest that these participants were not fully engaged while completing the task. The final sample for analyses of the selective attention task thus included 47 participants. All results from the mixed ANOVAs for the selective attention task are presented in **Table 2**.

**Table 2:** Results of the trial type × group × sex × task ANOVAs for the selective attention task.

Measure	Effect	*df*	*F*	*p*	η^2^
Accuracy	Trial type	1.1, 44.1	64.69	<0.001***	0.62
	Group	1, 39	1.12	0.296	0.03
	Sex	1, 39	5.11	0.029*	0.12
	Task order	1, 39	1.41	0.242	0.04
	Trial type × Group	1.1, 44.1	2.21	0.142	0.05
	Trial type × Sex	1.1, 44.1	6.69	0.011*	0.15
	Trial type × Task order	1.1, 44.1	1.51	0.229	0.04
	Group × Sex	1, 39	0.01	0.946	<0.01
	Group × Task order	1, 39	1.33	0.256	0.03
	Sex × Task order	1, 39	0.09	0.761	<0.01
	Trial type × Group × Sex	1.1, 44.1	0.02	0.919	<0.01
	Trial type × Group × Task order	1.1, 44.1	0.63	0.450	0.02
	Trial type × Sex × Task order	1.1, 44.1	0.17	0.717	<0.01
	Group × Sex × Task order	1, 39	0.65	0.424	0.02
	Trial type × Group × Sex × Task order	1.1, 44.1	2.17	0.145	0.05

Response Time	Group	1, 39	1.76	0.001**	0.23
	Sex	1, 39	0.10	0.754	<0.01
	Task order	1, 39	0.02	0.897	<0.01
	Trial type × Group	1.4, 53.1	0.69	0.455	0.02
	Trial type × Sex	1.4, 53.1	0.56	0.508	0.01
	Trial type × Task order	1.4, 53.1	1.60	0.215	0.04
	Group × Sex	1, 39	1.50	0.228	0.03
	Group × Task order	1, 39	1.01	0.321	0.03
	Sex × Task order	1, 39	0.14	0.713	<0.01
	Trial type × Group × Sex	1.4, 53.1	1.7	0.256	0.03
	Trial type × Group × Task order	1.4, 53.1	0.11	0.823	<0.01
	Trial type × Sex × Task order	1.4, 53.1	1.31	0.270	0.03
	Group × Sex × Task order	1, 39	0.01	0.937	<0.01
	Trial type × Group × Sex × Task order	1.4, 53.1	0.72	0.443	0.02

*^∗^p < 0.05, ^∗∗^p < 0.01, ^∗∗∗^p < 0.001.*

#### Accuracy

**FIGURE 4:**
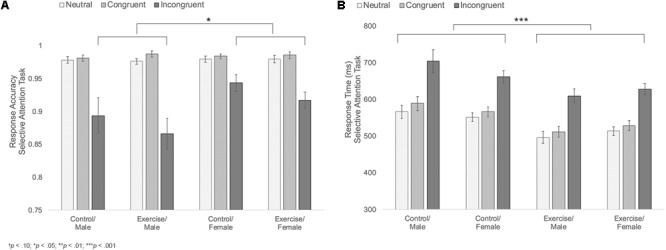
**(A)** Mean response accuracy during neutral, congruent, and incongruent trials of the selective attention task for male and female participants in the control versus exercise groups. **(B)** Mean response times during neutral, congruent, and incongruent trials of the selective attention task for male and female participants in the control versus exercise groups. Error bars represent standard error.

Results indicated main effects of trial type and sex [*F*_Trialtype_(1.1,44.1) = 64.69, *p* < 0.001, η^2^ = 0.62; *F*_Sex_(1,39) = 5.11, *p* = 0.029, η^2^ = 0.12], with participants showing the expected decline in accuracy during the incongruent trials and females showing overall higher accuracy compared to males. These main effects were moderated by a significant trial type × sex interaction, *F*(1.1,44.1) = 6.69, *p* = 0.011, η^2^ = 0.15; **Figure 4A**. Follow-up analyses indicated that females were more accurate than males during incongruent trials [*M*_F_ = 0.93, *SD* = 0.05; *M*_M_ = 0.88, *SD* = 0.08; *t*(29.9) = 2.62, *p* = 0.019, *d* = 0.74]. There were no differences in accuracy across the two groups during neutral or congruent trials [Neutral: *M*_F_ = 0.98, *SD* = 0.02; *M*_M_ = 0.98, *SD* = 0.02; *t*(45) = 0.42, *p* = 0.675; Congruent: *M*_F_ = 0.98, *SD* = 0.01; *M*_M_ = 0.98, *SD* = 0.01; *t*(45) = 0.18, *p* = 0.854]. There were no other significant effects (**Table 2**).

#### Reaction Time

Results indicated a significant main effect of trial type, *F*(1.4,53.1) = 296.21, *p* < 0.001, η^2^ = 0.88. Paired comparisons indicated that participants responded more slowly during incongruent trials (*M* = 650.90 ms, *SD* = 77.37 ms) relative to both congruent [*M* = 549.72 ms, *SD* = 58.59 ms; *t*(46) = 17.18, *p* < 0.001, *d* = 2.5] and neutral trials [*M* = 532.75 ms, *SD* = 54.83 ms; *t*(45) = 20.27, *p* < 0.001, *d* = 2.96]. Participants were also slower during congruent trials relative to neutral trials [*t*(46) = 6.09, *p* < 0.001, *d* = 0.89]. Thus participants showed the expected reaction time cost associated with the presence of conflicting distractors during incongruent trials.

Results also indicated a significant main effect of group, *F*(1,39) = 11.76, *p* = 0.001, η^2^ = 0.23, **Figure 4B**, with participants in the exercise group showing overall faster response times (*M*_Exercise_ = 548.20 ms, *SD* = 57.90 ms; *M*_Control_ = 605.75 ms, *SD* = 57.08 ms). Group did not interact with trial type, sex, or trial type x sex, indicating that male and female participants in the exercise condition showed similarly facilitated response times across all three trial types. No other effects reached significance (**Table 2**).

#### Continuous Analyses

We also examined whether individual differences in participants’ exertion during the activity protocols, as measured by HRR%, related to their accuracy or response times during the selective attention task. For each of these dependent variables we used multiple regression with HRR%, participant sex, task order, and the resulting interaction terms included as predictors. One female participant was excluded from these analyses because her HRR% was >2 *SD* above the group mean. All regression coefficients are presented in **Table 3**.

**Table 3:** Regression coefficients for the selective attention task.

	Overall accuracy		Overall response time	
Variable	*B* (*SE*)	*t*	*B* (*SE*)	*t*
Constant	0.95 (*0.01*)	105.02***	629.06 (*19.51*)	32.24***
HRR%	<0.001 (*<0.001*)	–0.18	–2.38 (*0.74*)	–3.20**
Sex	0.02 (*0.01*)	1.79^†^	–30.98 (25.74)	–1.20
Task Order	–0.01 (*0.01*)	–0.76	3.04 (19.51)	0.16
HRR% × Sex	<0.001 (*<0.001*)	–0.29	1.35 (0.94)	1.44
HRR% × Task Order	<0.001 (*<0.001*)	0.34	–0.35 (0.74)	–0.48
Sex × Task Order	–0.01 (*0.01*)	–0.51	1.07 (25.74)	0.04
HRR% × Sex × Task Order	<0.001 (*<0.001*)	0.69	–0.07 (0.94)	–0.07
*N*	45		45	
*R^2^*	0.22		0.29

*^†^*p* < 0.10, ^∗^*p* < 0.05, ^∗∗^*p* < 0.01, ^∗∗∗^*p* < 0.001.*

##### Response accuracy

Results indicated that sex was a marginally significant predictor of overall accuracy during the selective attention task, *B* = 0.02, *t*(37) = 1.79, *p* = 0.080. Consistent with the results reported above, female participants showed higher accuracy compared to males. HRR% was not related to performance during the selective attention task (**Table 3**).

##### Response time

Results indicated that only HRR% was a significant negative predictor of overall response time during the selective attention task, *B* = -2.38, *t*(37) = -3.20, *p* = 0.003. Consistent with the results reported above, participants who showed higher levels of exertion during the activity protocols also showed faster response times during the selective attention task.

### Memory Task

We used ANOVA with group (exercise, control), participant sex (male, female), and task order (attention-memory, memory-attention) as between subjects factors to examine (1) accuracy and response times during the encoding phase and (2) recognition memory sensitivity, object location memory sensitivity, and response times during the test phase of the task. Two participants (1 male control, 1 female control) were excluded from all analyses due to high rates of failing to respond during the encoding phase (>3 *SD* above the group mean). One additional participant (female exercise) was excluded from all analyses due to poor accuracy during the encoding phase (<3 *SD* below the group mean). Finally, one participant (female exercise) was excluded from all analyses due to a high rate of failing to respond during the test phase (>3 *SD* above the group mean). As in the selective attention task, these high rates of failing to respond or committing errors suggest that these participants were not fully engaged in the task. The final sample for analyses of the memory task thus included 48 participants.

#### Encoding Phase

Results of the ANOVAs for the encoding phase of the memory task are presented in **Table 4**.

**Table 4:** Results of the group × sex × task ANOVAs for the encoding phase of the memory task.

Measure	Effect	*df*	*F*	*p*	η^2^
Encoding	Group	1, 40	0.01	0.940	<0.01
accuracy	Sex	1, 40	0.45	0.498	0.01
	Task order	1, 40	2.55	0.118	0.06
	Group × Sex	1, 40	0.89	0.350	0.02
	Group × Task order	1, 40	1.79	0.189	0.04
	Sex × Task order	1, 40	0.63	0.432	0.02
	Group × Sex × Task order	1, 40	0.70	0.408	0.02

Encoding	Group	1, 40	2.05	0.160	0.05
response time	Sex	1, 40	0.07	0.788	<0.01
	Task order	1, 40	0.11	0.742	<0.01
	Group × Sex	1, 40	0.75	0.393	0.02
	Group × Task order	1, 40	0.32	0.578	<0.01
	Sex × Task order	1, 40	0.33	0.571	<0.01
	Group × Sex × Task order	1, 40	4.53	0.039^∗^	0.10

*^†^*p* < 0.10, ^∗^*p* < 0.05, ^∗∗^*p* < 0.01, ^∗∗∗^*p* < 0.001.*

##### Response accuracy

Results indicated no significant effects on accuracy during the encoding phase.

##### Response time

Results indicated a significant group × sex × task order interaction, *F*(1,40) = 4.53, *p* = 0.039, η^2^ = 0.10. Follow-up analyses indicated that there was a significant group × sex interaction among participants who completed the memory task second, *F*(1,20) = 6.50, *p* = 0.019, η^2^ = 0.25, but not among those who completed the memory task first, *F*(1,20) = 0.61, *p* = 0.443. Among the participants who completed the task second, there was a main effect of sex within the control group, *F*(1,9) = 6.17, *p* = 0.035, η^2^ = 0.41, with male participants responding faster than females during the encoding phase (*M*_M_ = 650.35 ms, *SD* = 161.92 ms; *M*_F_ = 879.16 ms, *SD* = 129.88 ms). There was no difference in response times for male and female participants in the exercise group, *F*(1,11) = 1.57, *p* = 0.237.

We also examined whether participants’ response times during the encoding phase of the memory task related to their recognition or object location memory sensitivity scores. Encoding response time was significantly positively related to both recognition memory scores [*r*(48) = 0.55, *p* < 0.001] and object location memory scores [*r*(48) = 0.40, *p* = 0.005]. Thus, participants who responded more slowly during the encoding phase of the memory task showed higher recognition and object location memory scores at test.

#### Memory Test Phase

Mean hit rates, false alarm rates, and *d*′ scores are presented in **Table 5**. Results of the ANOVAs for the test phase of the memory task are presented in **Table 6**.

**Table 5:** Mean hit rate, false alarm rate, and *d*′ scores for recognition and object location memory.

		Recognition memory sensitivity			Object location memory sensitivity		
		Hits	False alarms	*d*′	Hits	False alarms	*d*′
		Mean (*SD*)			Mean (*SD*)		
Control	Male	0.65 (*0.15*)	0.30 (*0.18*)	1.14 (*0.78*)	0.58 (*0.19*)	0.29 (*0.11*)	0.82 (0.83)
	Female	0.71 (*0.24*)	0.13 (*0.14*)	1.97 (*1.05*)	0.77 (*0.16*)	0.14 (*0.16*)	2.28 (*1.32*)
Exercise	Male	0.75 (*0.13*)	0.19 (*0.14*)	1.77 (*0.72*)	0.75 (*0.16*)	0.23 (*0.13*)	1.56 (*0.90*)
	Female	0.70 (*0.15*)	0.19 (*0.28*)	2.03 (*0.97*)	0.74 (*0.20*)	0.20 (*0.15*)	1.68 (1.17)

**Table 6:** Results of the group × sex × task ANOVAs for the test phase of the memory task.

Measure	Effect	*df*	*F*	*p*	η^2^
Recognition	Group	1, 40	1.84	0.182	0.04
memory	Sex	1, 40	5.75	0.021^∗^	0.13
	Task order	1, 40	1.13	0.294	0.03
	Group × Sex	1, 40	1.87	0.179	0.05
	Group × Task order	1, 40	0.35	0.555	<0.01
	Sex × Task order	1, 40	6.29	0.016^∗^	0.14
	Group × Sex × Task order	1, 40	2.74	0.105	0.06

Location	Group	1, 40	0.02	0.890	<0.01
memory	Sex	1, 40	7.92	0.008^∗∗^	0.17
	Task order	1, 40	1.38	0.247	0.03
	Group × Sex	1, 40	5.91	0.020^∗^	0.13
	Group × Task order	1, 40	0.06	0.804	<0.01
	Sex × Task order	1, 40	3.99	0.053^†^	0.09
	Group × Sex × Task order	1, 40	3.30	0.077^†^	0.08

Response	Group	1, 40	0.11	0.738	<0.01
time	Sex	1, 40	5.93	0.019^∗^	0.13
	Task order	1, 40	3.74	0.06^†^	0.09
	Group × Sex	1, 40	1.32	0.258	0.03
	Group × Task order	1, 40	2.70	0.108	0.06
	Sex × Task order	1, 40	0.16	0.692	<0.01
	Group × Sex × Task order	1, 40	1.45	0.236	0.04

*^†^p < 0.10, ^∗^p < 0.05, ^∗∗^p < 0.01, ^∗∗∗^p < 0.001.*

##### Recognition memory sensitivity (*d*′)

Results showed a significant main effect of sex, *F*(1,40) = 5.75, *p* = 0.021, η^2^ = 0.13, as well as a significant sex × task order interaction, *F*(1,40) = 6.29, *p* = 0.016, η^2^ = 0.14. Among those who completed the memory task second, female participants showed better recognition memory relative to male participants [*M*_F_ = 2.45, *SD* = 0.80, *M*_M_ = 1.35, *SD* = 0.75; *t*(22) = 3.49, *p* = 0.002, *d* = 1.43]. In contrast there was no difference across male and female participants’ recognition memory performance among participants who completed the task first [*M*_F_ = 1.55, *SD* = 0.99, *M*_M_ = 1.62, *SD* = 0.86; *t*(22) = 0.19, *p* = 0.851].

**FIGURE 5:**
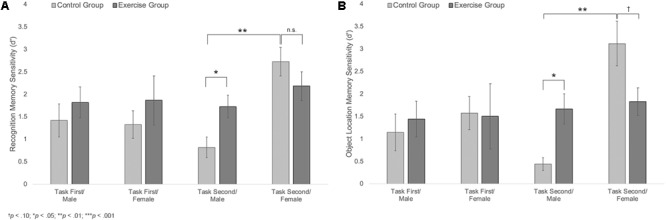
**(A)** Mean recognition memory sensitivity (*d*′) scores for male and female participants in the exercise and control groups. **(B)** Mean object location memory sensitivity scores (*d*′) for male and female participants in the exercise and control groups. Error bars represent standard error.

Results also showed a trending group × sex × task order interaction, *F*(1,40) = 2.74, *p* = 0.105, η^2^ = 0.14, **Figure 5A**. Follow-up analyses showed a significant group × sex interaction among participants who completed the memory task second, *F*(1,20) = 6.33, *p* = 0.021, η^2^ = 0.24, but not among those who completed the memory task first, *F*(1,20) = 0.03, *p* = 0.859. Among those who completed the task second, males in the exercise group showed significantly better recognition memory compared to those in the control group [*M*_Exercise_ = 1.73, *SD* = 0.66, *M*_Control_ = 0.81, *SD* = 0.52; *t*(10) = 2.55, *p* = 0.029, *d* = 1.55]. Females in the control group also showed significantly better recognition memory compared to males in the same group [*t*(10) = 4.65, *p* = 0.001, *d* = 2.88]. However, there were no differences in recognition memory performance across males and females in the exercise group [*t*(11) = 1.12, *p* = 0.285] or across female participants in the exercise and control groups [*M*_Exercise_ = 2.18, *SD* = 0.79, *M*_Control_ = 2.72, *SD* = 0.78; *t*(10) = 1.20, *p* = 0.258].

##### Object location memory sensitivity

Results indicated a significant main effect of sex, *F*(1,40) = 7.92, *p* = 0.008, η^2^ = 0.17, a significant group × sex interaction, *F*(1,40) = 5.91, *p* = 0.02, η^2^ = 0.13, and a marginally significant sex × task order interaction, *F*(1,40) = 3.99, *p* = 0.053, η^2^ = 0.09. These effects were moderated by a trend-level significant group × sex × task order interaction, *F*(1,40) = 3.30, *p* = 0.077, η^2^ = 0.08; **Figure 5B**. Additional analyses indicated that there was a significant group × sex interaction for participants who completed the memory task second, *F*(1,20) = 12.19, *p* = 0.002, η^2^ = 0.38, but not for those who completed the memory task first, *F*(1,20) = 0.15, *p* = 0.702. Among those who completed the memory task second, males in the exercise group showed significantly better object location memory compared to those in the control group [*M*_Exercise_ = 1.66, *SD* = 0.89, *M*_Control_ = 0.44, *SD* = 0.32; *t*(10) = 2.90, *p* = 0.016, *d* = 1.82]. Females in the control group also showed significantly better performance than males in the same group [*t*(9) = 5.20, *p* = 0.002, *d* = 3.03]. There was no difference in object location memory across male and female participants in the exercise group [*t*(11) = 0.36, *p* = 0.723]. However, females in the control group showed marginally better object location memory performance compared to females in the exercise group [*M*_Control_ = 3.12, *SD* = 1.21; *M*_Exercise_ = 1.83, *SD* = 0.75; *t*(10) = 2.22, *p* = 0.056, *d* = 1.28].

##### Response time

Results indicated a main effect of sex, *F*(1,40) = 5.93, *p* = 0.019, η^2^ = 0.13, with females showing overall faster responses during the test phase (*M*_F_ = 1284.36 ms, *SD* = 160.0; *M*_M_ = 1425.91 ms, *SD* = 226.83). No other effects reached significance (**Table 6**).

##### Continuous analyses

Last, we examined whether individual differences in participants’ exertion during the activity protocols, as measured by HRR%, were related to their recognition memory or object location memory scores. For each of these dependent variables we used multiple regression with HRR%, participant sex, task order, and the resulting interaction terms included as predictors. One female participant was excluded from these analyses because her HRR% was more than 2 *SD* above the group mean. All regression coefficients are presented in **Table 7**.

**Table 7:** Regression coefficients for the memory test phase.

	Recognition memory		Object location memory	
Predictor	*B* (*SE*)	*t*	*B* (*SE*)	*t*
Constant	1.04 (*0.29*)	3.58**	0.66 (*0.34*)	1.97^†^
HRR%	0.02 (*0.01*)	1.90^†^	0.02 (*0.01*)^†^	1.97^†^
Sex	1.11 (*0.38*)	2.93**	1.89 (*0.44*)***	4.26**
Task Order	0.38 (*0.29*)	1.30	0.52 (*0.34*)	1.55
HRR% × Sex	–0.03 (*0.01*)*	–2.10*	–0.05 (*0.02*)**	–3.23**
HRR% × Task Order	–0.01 (*0.01*)	–0.84	–0.02 (*0.01*)	–1.49
Sex × Task Order	–1.01 (*0.38*)*	–2.65*	–1.24 (*0.44*)**	–2.80**
HRR% × Sex × Task Order	0.02 (*0.01*)	1.39	0.03 (*0.02*)^†^	1.91^†^
*N*	46		46	
*R^2^*	0.30		0.41	

*^†^p < 0.10, ^∗^p < 0.05, ^∗∗^p < 0.01, ^∗∗∗^p < 0.001.*

*Recognition memory*. Results indicated that sex was a significant predictor of recognition memory, *B* = 1.11, *t*(38) = 2.93, *p* = 0.006, while HRR% was a trend-level significant predictor of recognition memory, *B* = 0.02, *t*(38) = 1.90, *p* = 0.065. Results also indicated a significant sex × task order interaction, *B* = -1.01, *t*(38) = -2.10, *p* = 0.042. Consistent with the results reported above, participant sex was a significant predictor of recognition memory for participants who completed the memory task second, *B* = 2.12, *t*(18) = 4.58, *p* < 0.001, with females showing better performance than males. Sex was not a significant predictor of recognition memory performance for participants who completed the memory task first, *B* = 0.11, *t*(20) = 0.19, *p* = 0.854.

**FIGURE 6:**
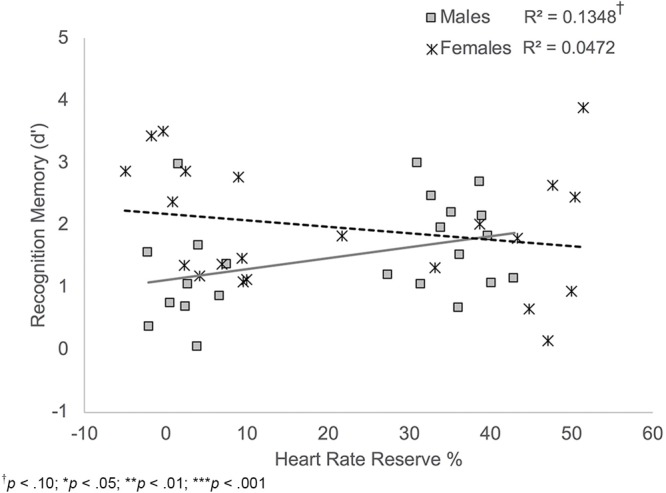
Scatterplot illustrating the interaction between HRR% and participant sex on recognition memory performance. Male participants who showed higher exertion showed better recognition memory compared to males who showed lower exertion. HRR% was not related to recognition memory performance among female participants.

Results also indicated a significant HRR% × sex interaction, *B* = 0.02, *t*(38) = -2.10, *p* = 0.042. Simple slopes analyses showed that there was a trend-level significant positive relationship between HRR% and recognition memory scores among male participants, *B* = -0.03, *t*(38) = 1.90, *p* = 0.065; **Figure 6**. Thus, males who showed increased exertion during the activity protocols showed better recognition memory performance. HRR% was not significantly related to recognition memory scores among female participants, *B* = -0.01, *t*(38) = -1.05, *p* = 0.298.

**FIGURE 7:**
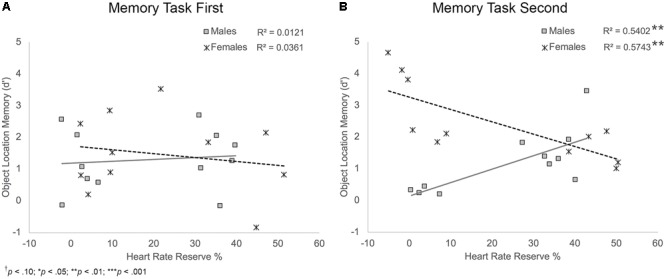
Scatterplots illustrating the interaction between HRR% and participant sex on object location memory performance for participants who completed the memory task first **(A)** and those who completed the memory task second **(B)**. Among those who completed the task second, males who showed higher exertion showed better object location memory compared to males who showed lower exertion. Females who showed higher exertion showed poorer object location memory compared to females who showed lower exertion.

*Object location memory*. Results showed that HRR% was a marginally significant predictor and sex was a significant predictor of object location memory, *B*_HRR_ = 0.02, *t*(38) = 1.97, *p* = 0.056; *B*_Sex_ = 1.89, *t*(38) = 4.26, *p* < 0.001. These main effects were moderated by significant HRR% × sex and sex × task order interactions, *B*_HRRxSex_ = -0.05, *t*(38) = -3.23, *p* = 0.003; *B*_SexxTO_ = -1.24, *t*(38) = -2.80, *p* = 0.008, and a trend-level significant HRR% × sex × task order interaction, *B* = 0.03, *t*(38) = 1.91, *p* = 0.064, **Figure 7**. Follow-up analyses showed that there was a significant HRR% × sex interaction for participants who completed the memory task second, *B* = -0.08, *t*(18) = -4.59, *p* < 0.001, but not for participants who completed the task first, *B* = -0.02, *t*(20) = -0.81, *p* = 0.430. For those who completed the task second, simple slopes analyses showed a significant positive relationship between HRR% and object location memory scores among male participants, *B* = 0.04, *t*(20) = 2.92, *p* = 0.009. Thus, males who completed the memory task second and showed greater exertion during the activity protocols showed better object location memory scores. Results also showed a significant negative relationship between HRR% and object location memory scores among female participants, *B* = -0.04, *t*(20) = -3.79, *p* = 0.001. Unlike males, females who completed the memory task second and showed greater exertion during the activity protocols showed poorer object location memory performance.

## Discussion

The present study demonstrates that both participant sex and acute, moderate-intensity exercise influence selective attention and memory performance among healthy, young adults. Critically, we utilized an active control design (Alves et al., 2014) to ensure that the observed cognitive benefits following acute exercise could not be attributed to expectation-driven placebo effects among participants in the exercise group. Furthermore we examined effects of exercise using both a categorical measure (i.e., exercise vs. control group) and by examining individual differences in exertion during the activity protocols, as indexed by heart rate reserve percentage (HRR%). These measures converged to show that acute exercise facilitated overall response times during the selective attention task, regardless of congruency, suggesting a general effect on processing speed. We additionally found sex differences in both selective attention and memory performance, with women showing higher accuracy during the incongruent trials of the attention task and better recognition and object location memory performance. Moreover, exercise differentially influenced men and women’s memory. Men who showed increased exertion during the activity protocols showed a modest benefit for recognition memory, whereas exertion was unrelated to women’s recognition memory performance. Exercise also differentially affected men’s and women’s object location memory, though only for participants who completed the memory task second. Within this group, men who showed increased exertion showed better object location memory performance, as for recognition memory. Women who showed increased exertion instead showed poorer object location memory performance but maintained an overall high level of performance.

These findings are consistent with previous research documenting benefits of acute aerobic exercise on young adults’ attention and memory skills (Kamijo et al., 2007; Coles and Tomporowski, 2008; Davranche and McMorris, 2009; Davranche et al., 2009; Labban and Etnier, 2011; Hopkins et al., 2012; Alves et al., 2014; Bullock and Giesbrecht, 2014; McNerney and Radvansky, 2015; Weng et al., 2015; Perini et al., 2016; Kao et al., 2017; Sng et al., 2017; Suwabe et al., 2017; Tempest et al., 2017). In particular, the current results replicate previous findings of an overall response facilitation during the Flanker task following acute moderate- or high-intensity exercise (Kamijo et al., 2007; Davranche et al., 2009; Weng et al., 2015; Kao et al., 2017; Tempest et al., 2017). The present study further showed that the extent of this response facilitation was related to participants’ exertion during the activity protocols. Both men and women who showed higher levels of exertion during the activity protocol responded faster during the selective attention task, regardless of whether they were in the exercise or control group.

The present findings are also consistent with prior research demonstrating enhanced recognition and object location memory among women (Voyer et al., 2007; Herlitz and Rehnman, 2008). It is important to note that all targets used in the current memory task were objects that could be easily labeled. Previous work suggests that the memory advantage observed among women may have been eliminated or reduced if we had instead used visuospatial stimuli or abstract objects that could not be easily identified with a verbal label (Lewin et al., 2001; Rahman et al., 2011). We also found a sex difference in performance during the selective attention task, with women showing higher accuracy than men during incongruent trials. This is in contrast to several previous studies that found poorer performance among women during the Flanker task (Stoet, 2010; Judge et al., 2012; Evans and Hampson, 2015). However, additional research has demonstrated the opposite effect, with women outperforming men on a Stroop selective attention task that similarly involves resolving conflict introduced by competing information (Mekarski et al., 1996; Van der Elst et al., 2006). Studies have also shown that women outperform men on tasks that require selective attention to peripheral or local stimuli (Roalf et al., 2006; Razumnikova and Volf, 2011) but this advantage can be influenced by hormonal changes associated with the menstrual cycle (Pletzer et al., 2014). The present study is limited in that we did not obtain data on female participants’ menstrual cycle phase when they completed the study. Given these mixed results, additional research is needed to clarify the nature of sex differences and the role of sex hormones in selective attention skills.

To our knowledge this is only the second study to examine differential effects of acute exercise on men and women’s cognitive function, with the previous study finding that 5 min of acute aerobic exercise following memory encoding improved women’s paired associate memory but did not affect men’s memory performance (Most et al., 2017). Unlike Most et al. (2017), the present study did not find an effect of exercise on recognition memory performance at the group level. This result was surprising given extensive previous research demonstrating that moderate-intensity aerobic exercise benefits recognition memory and discrimination of old and new items (Roig et al., 2013; McNerney and Radvansky, 2015; Suwabe et al., 2017). One possibility is that the exercise in the present study was not sufficiently intense to support improvements in recognition memory. On average, participants in the exercise group showed only moderate increases in heart rate reserve percentages (i.e., HRR%) following exercise. Although this was significantly higher than in the control group, participants in the exercise group may have had larger benefits for recognition memory if they had engaged in exercise that elevated their heart rate to a greater extent. In support, we found a relationship between participants’ HRR% and their recognition memory scores, indicating that participants who exerted more effort during the activity protocols had better recognition memory. However, we only observed this effect among male participants, suggesting that an exercise intervention that more effectively increases HRR% may specifically benefit men’s recognition memory performance.

It is also possible that the present study was not sufficiently powered to detect an effect of exercise on recognition memory. Previous empirical studies and meta-analyses examining the effects of acute aerobic exercise on memory have observed small effect sizes (see **Supplementary Table S1**). For example, studies examining main effects of participant sex or exercise on memory have observed effects ranging from η^2^ = 0.01 to η^2^ = 0.08. Most et al. (2017) examined interactive effects of sex and exercise on paired-associates memory with an observed effect size of η^2^ = 0.11. Relatively large sample sizes are required to detect these small effects. Power analyses indicated that the present study would require 66 participants to detect an effect size of η^2^ = 0.11 at power of 0.80. Thus the modest sample size in the present study (*N* = 48) may have limited our ability to detect a sex × exercise interaction effect on recognition memory. Additional studies with larger sample sizes will be needed to further examine how participant sex and acute exercise interact to influence recognition memory.

Although we did not observe an effect of exercise on recognition memory at the group level, we did find that exercise interacted with participant sex to influence object location memory. However, this effect was only evident among participants who completed the selective attention task first and the memory task second. Within this group, acute exercise prior to encoding improved men’s object location memory. Importantly, this cannot be attributed to greater efficacy of the exercise intervention among men, as women showed overall higher HRR% compared to men. Women showed an overall high level of object location memory performance, but exercise instead had a negative effect on their object location memory. However, there was no difference in object location memory across men and women in the exercise group, suggesting that the negative effect of exercise on women’s object location memory may be attributed to superior performance among women in the control group. These women also showed slower response times during the memory encoding phase, which may reflect deeper encoding that ultimately supported better memory for the initial target location.

These results contrast with Most et al. (2017), which found a paired-associates memory advantage among women following acute exercise. One key difference between these two studies is the timing of exercise relative to memory encoding. In the present study the exercise protocol was completed prior to memory encoding, whereas exercise was completed after encoding in Most et al. (2017), during the period of memory consolidation. Exercise thus likely modulated different memory mechanisms (i.e., encoding versus consolidation) across these two studies, which may have contributed to different performance outcomes for men and women. Future studies will be needed to tease apart differential effects of acute exercise for men and women during memory encoding, consolidation, and retrieval.

It is also important to note that recognition memory and object location memory may be supported by different subregions in the medial temporal lobe. Multiple fMRI studies have shown that successful recognition memory is associated with reduced activation in perirhinal cortex (Yonelinas et al., 2001; Weis et al., 2004; Slotnick, 2013) whereas memory for contextual information (i.e., source memory) is associated with increased activity in the hippocampus and parahippocampus (Yonelinas et al., 2001; Giovanello et al., 2004; Ross and Slotnick, 2008; Slotnick, 2013; Park et al., 2014). For example, Ross and Slotnick (2008) examined functional activity in medial temporal lobe regions using an object location memory task similar to the one used in the present study, with objects presented in left vs. right locations during encoding. Object location memory (i.e., identifying the left vs. right encoding context), but not recognition memory, was related to increased activity in bilateral hippocampus and left parahippocampus (Ross and Slotnick, 2008). Given extensive previous research indicating that increased hippocampal neurogenesis may mediate the relationship between aerobic exercise and improved learning and memory (van Praag et al., 2002; Farioli-Vecchioli and Tirone, 2015), these regional variations in medial temporal lobe activity may be important for understanding how exercise differentially impacts recognition memory and contextual memory processes.

As described above, the present study also found that the effect of participant sex on memory performance was moderated by the order in which participants completed the two cognitive tasks. Specifically, men and women who completed the memory task after the second activity protocol showed differential memory performance. Women in the control group responded more slowly during encoding and showed better object location memory compared to all other groups, while men in the exercise group showed improved object location memory compared to men in the control group. In contrast, there were no differences in men and women’s memory performance among participants who completed the task after the first activity protocol. Overall, there was no difference in participants’ mean HRR% across the first and second activity protocol, indicating that this effect of task order cannot be attributed to a simple increase in exertion over the course of the test session. Speculatively, completing the memory task after the attention task or the second activity protocol may have influenced participants’ behavior during the encoding phase of the of the memory task. In particular, among participants who completed the memory task second, men in the control group responded faster during the encoding phase compared to women in the same group, whereas there were no differences in response times across men and women in the exercise group. Encoding response times may reflect the depth of processing during encoding, with slower response times reflecting deeper encoding. These differences in encoding response times among men and women in the control group who completed the memory task second may thus have contributed to the differences in memory performance observed at test. The present study examined participants’ performance following exercise but did not evaluate baseline performance on the cognitive tasks prior to the activity protocols. As such it is difficult to know whether the differential performance among participants who completed the memory task second was due to the additional exertion of the second activity protocol or the experience of completing the selective attention task first. Future research should integrate the present exercise/active control design with within-subject comparisons of cognitive performance before and after exercise to further understand these dynamics.

The present behavioral data cannot speak to the underlying mechanisms that link acute exercise to the observed differences in selective attention and memory performance. Previous studies have suggested that improvements in cognitive function following acute exercise may be mediated by increases in regional blood flow (Endres et al., 2003; Pereira et al., 2007), hippocampal neurogenesis (van Praag et al., 2002), and/or release of neurotrophins (Neeper et al., 1995). One neurotrophin in particular, BDNF, is critical for neural plasticity and learning and memory (van Praag et al., 2002; Phillips et al., 2014). BDNF levels are also regulated by physical activity, with moderate-intensity acute exercise eliciting increased BDNF levels among young adults (Gold et al., 2003; Vega et al., 2006; Ferris et al., 2007; Tang et al., 2008; Heyman et al., 2012; Tsai et al., 2014). Researchers have proposed that this increased BDNF following exercise may mediate the beneficial effects of exercise on cognition by promoting neural plasticity and hippocampal neurogenesis (Voss et al., 2013). In support, several studies have found that individual differences in BDNF levels following exercise predicted the extent of learning and memory improvement (Winter et al., 2007; Ruscheweyh et al., 2011; Heisz et al., 2012) as well as structural changes in brain regions involved in attention and memory (Ruscheweyh et al., 2011).

Exercise may also interact with sex hormones to differentially affect cognitive performance among men and women. Exercise has been related to increased levels of circulating sex hormones (i.e., estrogen, testosterone) (Janssen, 2016) as well as increased local synthesis of these hormones both peripherally (Aizawa et al., 2007) and in the central nervous system (Okamoto et al., 2012). Evidence also suggests that sex hormones interact with BDNF, with increases in estrogen or estradiol eliciting greater BDNF expression in the cortex and hippocampus of females (Sohrabji et al., 1995; Scharfman et al., 2003). Speculatively, it is thus possible that the women in the present study had relatively high levels of BDNF due to circulating estrogens, contributing to their overall higher level of recognition and object location memory performance. In contrast, men in the control group may have had relatively lower BDNF levels, contributing to poorer memory performance. The exercise protocol may have elevated BDNF levels among men, resulting in object location memory performance that was better relative to men in the control group and equal to that of the women. The present study is limited in that we did not directly measure levels of BDNF or sex hormones or control for the timing of female participants’ hormonal cycles. Future studies will be necessary to more carefully examine the precise neurobiological mechanisms underlying differential effects of exercise on memory performance across men and women.

Overall, the current results converge with prior studies demonstrating that moderate-intensity acute exercise influences subsequent cognitive performance and with those showing overall enhanced memory among women. Consistent with this previous work, the present study found that exercise was associated with faster response times during the selective attention task and that women demonstrated better recognition and object location memory performance compared to men. Furthermore, exercise moderated the effect of participant sex on object location memory among participants who completed the memory task second, as men in the exercise group showed improved performance over those in the control group and similar memory performance compared to women in either group. Animal models and human physiological studies suggest that the specificity of this effect may be related to differential interactions between exercise, BDNF, neural networks supporting recognition and contextual memory, and sex hormones across men and women. This is only the second study (c.f., Most et al., 2017) to examine interactions between participant sex and acute exercise on memory performance. Future multi-method studies with sample sizes that offer sufficient power to examine these complex interactions will be needed to fully understand the neural mechanisms driving differential effects of exercise on selective attention and memory across men and women.

## Ethics Statement

This study was carried out in accordance with the recommendations of the Investigator Guidelines provided by the Institutional Review Board at Tulane University. The protocol was approved by the Tulane University Biomedical Institutional Review Board. All subjects gave written informed consent in accordance with the Declaration of Helsinki.

## Data Availability

The datasets for this manuscript are not publicly available due to local Institutional Review Board restrictions. Requests to access the datasets should be directed to Julie Markant at jmarkant@tulane.edu.

## Author Contributions

MC and JM contributed to the conception and design of the study. MC, KO, and JM prepared the stimuli and experimental tasks. MC and KO recruited and tested participants. MC and JM analyzed the data and wrote the first draft of the manuscript. All authors contributed to manuscript revision, read and approved the submitted version.

## Conflict of Interest Statement

The authors declare that the research was conducted in the absence of any commercial or financial relationships that could be construed as a potential conflict of interest.
