# Supplementary material for: Exercise Similarly Facilitates Men and Women’s Selective Attention Task Response Times but Differentially Affects Memory Task Performance
Source: Front Psychol. 2018 Aug 13;9:1405. doi: 10.3389/fpsyg.2018.01405 (PMC6100625; doi:10.3389/fpsyg.2018.01405)
Supplement: Supplementary file 1 [file Table_1.pdf]

Table S1. Effects sizes observed in previous studies of sex and exercise on selective attention and memory, the sample size needed to observe a similar effect size at power = .8, and the power of the current study to observe a similar effect size at power = .8.

	Observed effect	Observed effect size (h_p^2)	N for power = .8	Current power
**Selective attention (N =47)**				
Davranche et al. (2009)	Main effect of exercise	.63	8	1.0
Kao et al. (2017)	Main effect of exercise	.08	64	.67
Clayson et al. (2011)	Main effect of sex	.06	80	.55
Clayson et al. (2011)	Sex x trial type	.01	482	.12
Evans & Hampson (2015)	Main effect of sex	.11	44	.84
Evans & Hampson (2015)	Sex x trial type	.04	120	.38
**Memory (N = 48)**				
Smith et al. (2010) (meta-analysis)	Main effect of exercise	.004	1909	.07
Roig et al. (2013) (meta-analysis)	Main effect of exercise	.08	92	.52
Heisz et al. (2013)	Main effect of sex	.08	92	.52
**Most et al. (2017)**	**Sex x exercise**	**.11**	**66**	**.66**
Voyer et al. (2007) (meta-analysis)	Main effect of sex (recognition memory)	.01	683	.11
Voyer et al. (2007) (meta-analysis)	Main effect of sex (location memory)	.02	347	.18
